# Supplementary material for: Vision-language model-based semantic-guided imaging biomarker for lung nodule malignancy prediction
Source: J Biomed Inform. Author manuscript; Available in PMC 2026 Jun 21. (PMC13283286; doi:10.1016/j.jbi.2025.104947)
Supplement: 1 [file NIHMS2185403-supplement-1.pdf]

Appendix A. Supplementary data

Appendix A.1. Semantic Features Annotated

Table A1. **Semantic Features Annotated by Radiologists for 1,261 Nodules from NLST Data.** We reported all semantic features and the percentage distribution of each class within these features for the entire NLST dataset annotated by radiologists at UCLA Health. Multiple margin characteristics may be annotated for a single nodule.

**Feature**	**Classes (%)**
**General Features**	
Longest Axial Diameter	3.8-10mm (60.6); 10-20 (31.2); 20-30 (7.1); 30-40 (0.7); 40-50 (0.2); 50-59.3 (0.2)
Short Diameter	2.0-10mm (79.4); 10-20 (18.4); 20-30 (1.6); 30-40 (0.5); 40-48.8 (0.1)
Nodule Margin	Smooth (65.9); Lobulated (23.2); Spiculated (24.7); Ill-defined (20.6); Notched (0.2); N/A (2.0)
Nodule Consistency	Peri-cystic (1.9); Solid (70.0); Pure ground glass (9.4); Semiconsolidation (8.0); Part-solid (10.6); N/A (0.1)
Nodule Shape	Irregular (37.3); Ovoid (31.6); Polygonal (11.3); Round (18.9); N/A (0.9)
Nodule Margin Conspicuity	Well marginated (80.8); Poorly marginated (18.1); N/A (1.1)
**Internal Features**	
Nodule Reticulation	Present (87.1); Absent (12.5); N/A (0.4)
Cyst-like Spaces	Present (13.8); Absent (85.6); N/A (0.6)
Necrosis	Present (0.4); Absent (99.6)
Eccentric Calcification	Present (3.2); Absent (96.4); N/A (0.4)
Cavitation	Present (0.4); Absent (99.6)
Intra-nodular Bronchiectasis	Present (3.0); Absent (96.8); N/A (0.2)
Airway Cutoff	Present (2.5); Absent (25.7); N/A (71.8)
**External Features**	
Vascular Convergence	Present (12.8); Absent (86.8); N/A (0.4)
Pleural Retraction	Present (21.7) - Mild and Obvious Dimpling; Absent (78.1); N/A (0.2)
Pleural Attachment	Present (51.5); Absent (48.2); N/A (0.3)
Paracicatricial Emphysema	Present (11.1); Absent (88.5); N/A (0.4)
Septal Stretching	Present (67.3); Absent (32.5); N/A (0.2)
**General Assessment**	
Level of Suspicion of Lung Cancer	Very Low (30.1); Moderately Low (22.6); Intermediate (20.3); Moderately High (12.8); High (13.4); N/A (0.8)

Appendix A.2. Text Generation from Tabular Data Using Gemini

Prompt:

> Think as if you are a radiologist, you have a table/dictionary for lung nodule evaluation, and you want to transform it into radiology reports. Here is the dictionary {...}. Just show me the report. Do not add any additional information. In English! You should have an impression and findings part. Findings are bullet points listing each of the features separately. These features should be in random order. Combine nodule margins and additional nodule margins. If there is a missing value, just ignore it. For impression, do a quick summary of the findings in sentence and then state the suspiciousness of lung cancer. You can mention the absences in the findings but do not mention those in the impression.

Example:

Findings:
- Axial location: Central
- Longest axial diameter (mm): 17.0
- Short diameter (mm): 5.1
- Nodule margins: Ill-defined, Spiculated, Serrated
- Nodule shape: Complex, Irregular
- Nodule consistency: Part-solid
- Nodule reticulation: Present
- Cyst-like spaces: Absent
- Intra nodular bronchiectasis: Absent
- Necrosis: Absent
- Cavitation: Absent
- Eccentric calcification: Absent
- Airway cutoff: Absent
- Pleural attachment: Present
- Pleural retraction: Mild dimpling
- Vascular convergence: Present
- Septal stretching: Present
- Paracicatricial emphysema: Absent

Impression:
A 17.0 × 5.1 mm, central, part-solid nodule is identified. The nodule demonstrates complex, spiculated margins and is associated with pleural attachment, mild dimpling, vascular convergence, and septal stretching. The level of suspicion for lung cancer is moderately high.

Appendix A.3. LIDC and NLST Semantic Features Harmonization

Table A2. **Harmonization of LIDC and NLST semantic features.** We consulted radiologists at our institution to map the semantic features in LIDC to the corresponding semantic features in our internal NLST database. Semantic features present in our in-house data but absent in LIDC are considered missing and were excluded during text generation.

**LIDC Semantic Features**	**Radiologists' Entry**	**Mapped to NLST Semantic Features**
Internal Structure	"Air"	Cyst-like spaces = "Present"
	All Others	Cyst-like spaces = "Absent"
Calcification	"Non central appearance"	Eccentric Calcification = "Present"
	All Others	Eccentric Calcification = "Absent"
Sphericity	> 3	Nodule Shape = "Round"
	≤ 3	Nodule Shape = "Ovoid"
Margin	≥ 3	Nodule Margin Conspicuity = "Well marginated"
	< 3	Nodule Margin Conspicuity = "Poorly marginated"
Lobulation	≥ 3	Nodule Margins = "Lobulated"
Spiculation	≥ 3	Nodule Margins = "Spiculated"
Texture	> 4	Nodule Consistency = "Solid"
	$= 2, 3, 4$	Nodule Consistency = "Part-solid"
	< 2	Nodule Consistency = "Pure ground glass"

Appendix A.4. Model Performance Removing Cases Failed in DeepLungIPN

To ensure a fair comparison with DeepLungIPN, which failed to predict lung cancer risk for certain cases, we removed the failed cases and reported AUROC and AUPRC scores in Table A3 and FPR and Precision at different recall levels in Table A4.

Table A3. **Model Performance on Lung Cancer Prediction Within One Year.**

**Models**	**NLST Test Set** **NLST** **N=186 (43)**		**External Datasets** **LUNGx** **69 (35)**		**DLCS** **838 (94)**		**UCLA** **51 (28)**	
	**AUROC**	**AUPRC**	**AUROC**	**AUPRC**	**AUROC**	**AUPRC**	**AUROC**	**AUPRC**
Imaging Only Models								
Sybil	-	-	0.660 [0.523,0.790]	0.670 [0.503,0.850]	0.798 [0.743,0.849]	0.472 [0.365,0.576]	0.734 [0.585,0.865]	0.780 [0.610,0.906]
Venkadesh et al.	-	-	0.675 [0.543,0.800]	0.703 [0.538,0.840]	-	-	-	-
Imaging + Semantic Features Models								
DeepLungIPN	0.862 [0.794,0.920]	0.679 [0.522,0.815]	0.709 [0.577,0.835]	0.658 [0.496,0.856]	0.851 [0.803,0.894]	**0.543** [0.436,0.645]	0.624 [0.458,0.779]	0.601 [0.435,0.821]
Ours (CLIP)	**0.903** [0.845,0.951]	**0.779** [0.638,0.884]	**0.764** [0.641,0.876]	**0.814** [0.680,0.912]	**0.862** [0.821,0.899]	0.498 [0.389,0.604]	**0.778** [0.643,0.898]	**0.823** [0.661,0.935]

Table A4. **False Positive Rate and Precision at a Given Recall.**

**Models**	**Recall ≈ 0.6**		**Recall ≈ 0.7**		**Recall ≈ 0.8**		**Recall ≈ 0.9**	
	**FPR↓**	**Precision↑**	**FPR↓**	**Precision↑**	**FPR↓**	**Precision↑**	**FPR↓**	**Precision↑**
**NLST Test Set**								
DeepLungIPN	0.070	0.667	0.148	0.588	0.190	0.486	0.430	0.386
Ours (CLIP)	**0.035**	**0.812**	**0.092**	**0.638**	**0.141**	**0.630**	**0.331**	**0.419**
**LUNGx**								
Sybil	0.235	0.724	0.294	0.615	0.735	0.483	0.912	0.500
Venkadesh et al.	0.382	0.600	0.441	0.571	0.618	0.560	**0.676**	**0.542**
DeepLungIPN	0.206	0.724	0.265	0.706	**0.471**	**0.596**	0.882	0.492
Ours (CLIP)	**0.088**	**0.875**	**0.176**	**0.774**	0.500	0.560	0.765	**0.542**
**DLCS**								
Sybil	0.132	0.368	0.223	0.281	0.438	0.193	0.578	0.157
DeepLungIPN	**0.097**	**0.424**	**0.145**	0.361	**0.224**	0.301	0.477	0.193
Ours (CLIP)	0.108	0.412	0.151	**0.363**	0.228	**0.305**	**0.378**	**0.208**
**UCLA**								
Sybil	**0.217**	**0.773**	0.391	0.690	0.609	0.622	0.652	**0.634**
DeepLungIPN	0.304	0.586	0.522	0.588	0.609	0.611	0.696	0.581
Ours (CLIP)	**0.217**	0.739	**0.261**	**0.769**	**0.261**	**0.733**	**0.565**	0.568

Appendix A.5. Result of Ablation Studies: P-values

Table A5. **Hypothesis Testing P-values in Ablation Studies.** This table shows the p-values for hypothesis testing performed in ablation studies. The * superscript denotes a statistically significant difference at a significance level of 0.05. To compare more than three models, we used the Friedman Chi-Square test with a post-hoc Nemenyi test. For comparing two models, we conducted the Wilcoxon signed-rank test. It is important to note that since we only have five samples, one from each fold, the lowest p-value for the Wilcoxon signed-rank test is 0.063, so it will never yield a significant result. Abbreviations: F - Fine-Tuning; P - Probe-Tuning; L - Low-Rank Adaptation; S - Logistic regression model trained on semantic features; V - CLIP vision encoder trained with imaging only; CLIP - Contrastive Language–Image Pre-training.

	**NLST Test Set**		**External Datasets**					
	**NLST N=188 (43)**		**LUNGx 70 (35)**		**DLCS 856 (94)**		**UCLA 51 (28)**	
	**AUROC**	**AUPRC**	**AUROC**	**AUPRC**	**AUROC**	**AUPRC**	**AUROC**	**AUPRC**
**CLIP Training With Different Tuning Methods**								
F vs P	0.609	0.254	0.139	0.254	0.139	0.139	0.139	0.139
F vs L	0.012*	0.004*	0.012*	0.004*	0.012*	0.012*	0.012*	0.012*
P vs L	0.139	0.254	0.609	0.254	0.609	0.609	0.609	0.609
**Training With Different Modalities**								
S vs V	0.069	0.012*	-	-	-	-	-	-
S vs CLIP	0.946	0.609	-	-	-	-	-	-
V vs CLIP	0.031*	0.139	0.063	0.063	0.063	0.125	0.063	0.063

Appendix A.6. Error Analysis: More Examples

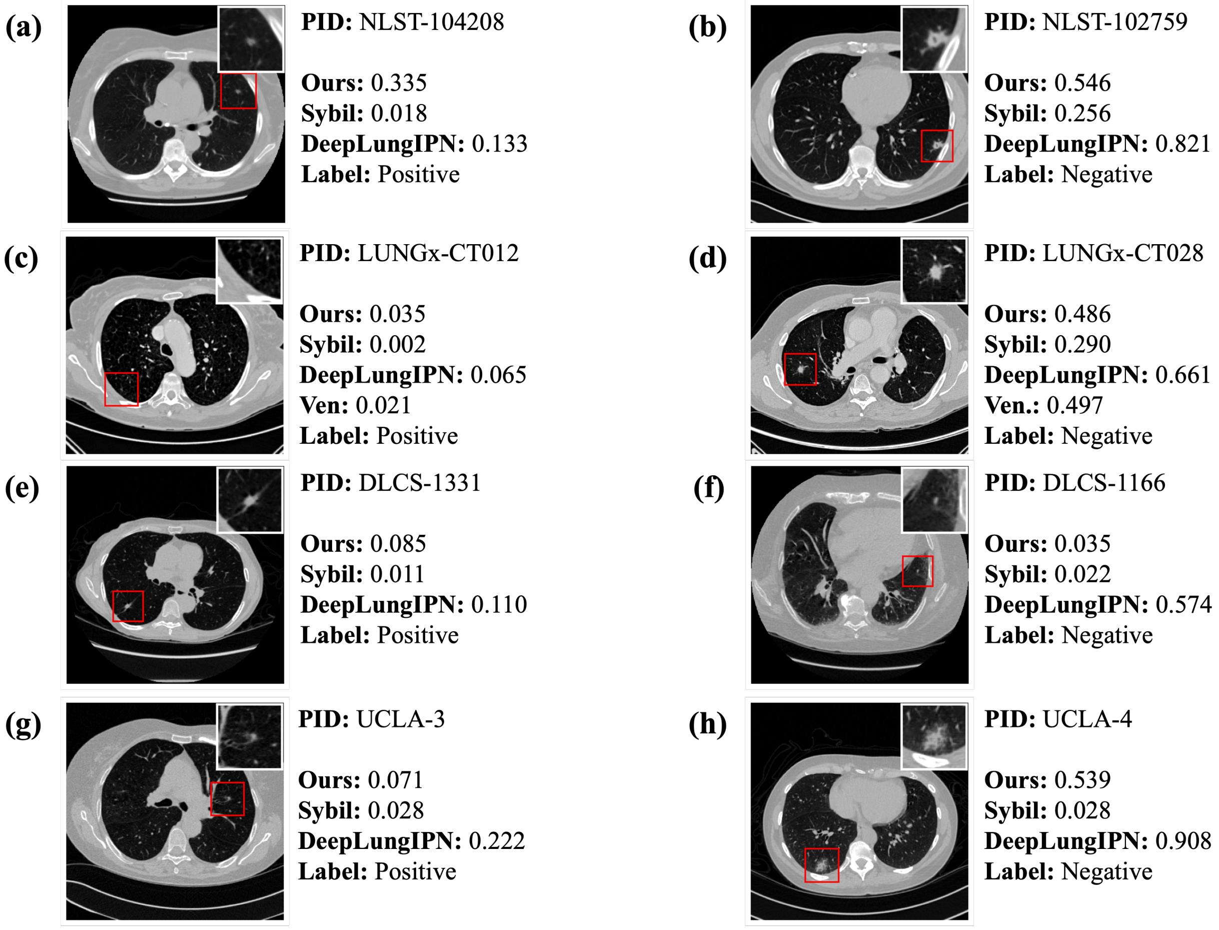

Fig. A1. **Error Analysis (Extended).** One lung cancer and one non-lung cancer case from each dataset are presented. We present the CT scan slice corresponding to the middle of the nodule, highlighted with a red bounding box and magnified in the top right corner. Certain features may not be fully appreciable in the single slice but are visible when viewing the whole series. Patient-level risk scores are shown beside images.

Fig. A1c, e, and g depict nodules diagnosed as lung cancer, but our model underestimates the risk. Although these nodules are relatively small, they exhibit irregular shapes (c), spiculated margins (e), mixed densities (g), and pleural attachment (c, e, g). Interestingly, all other models also predict low scores for these nodules. In Fig. A1a, a lobulated, solid, well-marginated nodule is shown with a few suspicious features. It is reasonable that our model predicts a 33% likelihood of malignancy, but all other models assign extremely low scores to this cancer patient.

In Fig. A1b, the nodule is solid with spiculated and lobulated margins and an irregular shape. Concerning characteristics include cyst-like spaces and pleural retraction. In Fig. A1d, the nodule appears to be solid with spiculated margins, septal stretching, and vascular convergence. In Fig. A1h, a large, part-solid nodule with poorly defined margins is observed.

Given these suspicious features, most models assign relatively higher risk scores, despite the patient not being diagnosed with lung cancer. However, in Fig. A1h, Sybil accurately identifies it as having a lower score. In contrast, Fig. A1f shows a tiny nodule with no apparent suspicious features, but DeepLungIPN assigns a comparatively higher risk. We found that the nodules detected by DeepLungIPN appear to be fibrosis and vessels.
